# Incidence of ADHD diagnoses and medication initiation among children and adolescents in Norway from 2016-2024

**DOI:** 10.1007/s00787-026-03116-1

**Published:** 2026-06-29

**Authors:** Saima Orangzeb, Ingeborg Hartz, Inger Johanne Bakken, Ingvild Odsbu, Kari Furu, Svetlana Skurtveit, Per Normann Andersen, Elisabeth Haug, Jacqueline M. Cohen

**Affiliations:** 1 Department of Chronic Diseases, Norwegian Institute of Public Health, Oslo, Norway; 2 Department of Research and Innovation, Innlandet Hospital Trust, Brumunddal, Norway; 3 Norwegian Centre for Addiction Research, University of Oslo, Oslo, Norway; 4 Department of Health and Nursing Sciences, University of Inland Norway, Rena, Norway; 5 Centre for Fertility and Health, Norwegian Institute of Public Health, Oslo, Norway; 6 Department of Psychology, University of Inland Norway, Lillehammer, Norway; 7 Department of Health Sciences, NTNU-Norwegian University of Science and Technology, Gjøvik, Norway

**Keywords:** Attention-Deficit/Hyperactivity Disorder, Incidence, Medication initiation, Pharmacotherapy, Norway, Children and adolescents, Register-based study

## Abstract

Global trends show an increase in ADHD diagnoses and medication use. This study aimed to describe the incidence of ADHD among children and adolescents in Norway from 2016 to 2024, and to investigate sociodemographic factors associated with treatment initiation among newly diagnosed individuals. This nationwide longitudinal study used data from Norwegian health registers, including all incident ADHD cases (first diagnosis or medication) at ages 3–17 years between 2016 and 2024. Incidence rates were calculated per 1,000 individuals along with proportion initiating medication 0–3 months and 4–12 months post diagnosis. Logistic regression models were used to assess factors associated with initiation. The overall incidence of ADHD more than doubled between 2016 and 2024 (from 4.4 to 9.0 per 1,000), with 52,149 incident ADHD cases. The most pronounced increase was observed among adolescent females (14–17 years), where incidence rose from 3.1 to 11.4 per 1,000. Among 38,749 individuals diagnosed between 2016 and 2023, the proportion initiating medication within one year of diagnosis increased from 72.8% to 78.4%. Following a peak in 2021, proportion initiation within 0–3 months declined, while initiation at 4–12 months increased. Having a foreign-born parent was associated with lower odds, while older age at diagnosis and lower paternal education associated with higher odds of medication-initiation. This study documents a doubling in the incidence of ADHD diagnoses among children in Norway, and an even stronger increase among adolescent females. Medication initiation increased slightly but evolved toward a delayed approach. Sociodemographic factors, especially age at diagnosis, parental region of birth and father’s education influence medication-initiation.

**Supplementary Information:**

The online version contains supplementary material available at https://doi.org/10.1007/s00787-026-03116-1.

## Introduction

Attention-Deficit/Hyperactivity Disorder (ADHD) is a prevalent neurodevelopmental disorder, characterized by symptoms of inattention, hyperactivity, and impulsivity that typically emerge in childhood [1]. In recent decades, the global prevalence of ADHD diagnoses and the subsequent consumption of medication have risen significantly [2, 3]. Norway shows this trend, with a marked increase in the dispensing of ADHD medications over the past decade [4]. This pattern highlights a pressing need to understand the factors, such as evolving diagnostic criteria, improved recognition, and societal pressures, that are contributing to this increase.

In Norway, the prevalence of ADHD medication uses among children aged 6 to 17 increased from 16 to 30 per 1,000 children between 2006 and 2022 [4]. A notable shift within this trend is the narrowing sex gap, with the male-to-female ratio dropping from 3.2 to 2.0, due to a recent rise in medication use among females. A comparative study of the Scandinavian countries found that from 2010 to 2020, ADHD medication use among children aged 5–19 years increased, with Sweden having the highest usage in 2020 (35 per 1,000) and the largest increase from 2010 (119%) as compared to Denmark (38%) and Norway (16%) [5].

Globally, the landscape of ADHD diagnosis and treatment is highly variable [2]. Beyond overall prevalence, international research highlights critical disparities in ADHD diagnosis and treatment linked to age, sex, and sociodemographic factors [6]. Age is a key factor, with diagnosis rates typically highest in early adolescence [2]. Furthermore, sociodemographic factors are strongly associated with ADHD diagnosis and management, for instance, lower parental education and income have been linked to severity of ADHD symptoms [7]. However, they are less likely to be diagnosed, potentially due to differential access to care or health-seeking behaviors [8–10].

While existing register studies have effectively documented the rise in ADHD medication use in Norway [4, 11], there is a gap in population-based data focusing specifically on the incidence of new diagnoses. Understanding this initial diagnosis is important, as it is the gateway to all subsequent treatment. Furthermore, prior research examining how the decision to initiate medication after a new diagnosis is influenced by child’s age, sex, and family sociodemographic background is limited.

This study has two main aims. First, to describe the trends in new ADHD diagnoses from 2016 to 2024 in Norwegian children and adolescents (3–17 years), looking at differences by age and sex. Second, to investigate patterns of medication initiation among newly diagnosed individuals, over time and by sociodemographic and parental factors.

## Methods

This observational study was based on linked individual-level data from nationwide health and population registers. We used data from the Norwegian Patient Registry (NPR) from inception in 2008, the Norwegian Prescribed Drug Registry (NorPD) from inception in 2004 and Statistics Norway [12–15]. NPR covers all publicly funded specialist outpatient and inpatient care in Norwegian hospitals but does not capture diagnoses made in privately funded healthcare settings without public contracts. NorPD records all prescribed drugs dispensed at pharmacies in Norway, meaning only medications that are both prescribed and collected by patients are included.

### Study population

The study population included all individuals born in Norway between 1998 and 2021 who were aged 3 to 17 at any time between 2016 and 2024. To be included, individuals must have received a diagnosis of ADHD (ICD-10 code F90) or have been dispensed an ADHD medication including amphetamine (N06BA01), dexamphetamine (N06BA02), methylphenidate (N06BA04), atomoxetine (N06BA09), lisdexamfetamine (N06BA12), or guanfacine (C02AC02). An additional inclusion criterion was at least one full year of Norwegian residency prior to the first ADHD diagnosis or medication dispensing, to exclude individuals arriving in Norway with an existing diagnosis from being counted as incident cases.

### ADHD incidence rate

**Fig. 1:**
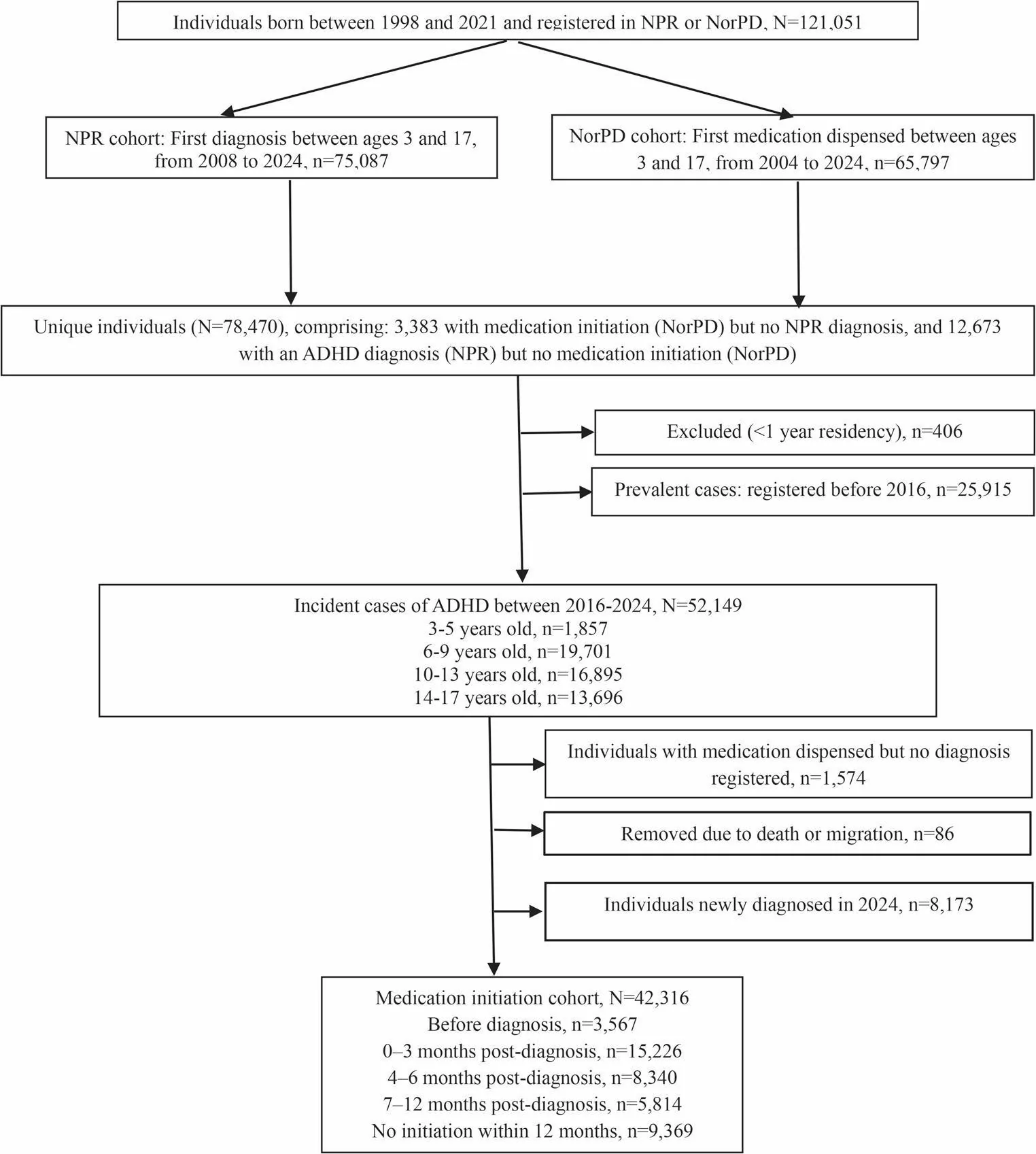
Identification of the study population. Incidence of ADHD (first ever diagnosis or dispensed ADHD medication) was described from 2016 to 2024 while medication initiation was described from 2016 to 2023, allowing for a year of follow-up after diagnosis. The ADHD medication initiation cohort comprises incident cases with ≥ 1 year of follow-up who had an NPR-registered diagnosis prior to medication initiation; individuals with dispensed medication but no corresponding diagnosis were excluded. The group initiating medication before diagnosis (*n* = 3567) was excluded from analyses of timing of initiation after diagnosis (remaining cohort: *N* = 38,749). NPR, National Patient Registry; NorPD, Norwegian Prescribed Drug Registry

We defined incident ADHD (Fig. 1) as the first date of either a visit in specialist healthcare with a recorded ADHD diagnosis code or date of the first dispensing of ADHD medication, whichever came first. We considered those with a diagnosis or dispensing at age 3 or older before 2016 to be prevalent at the start of the study period. Each individual is counted only once as an incident case.

For each year, the number of incident cases were identified. The population at risk was derived from Statistics Norway by starting with the total population (3–17-year-olds) as of December 31st for that specific year and subtracting the number of individuals aged 3–17 years who were diagnosed with ADHD prior to the year of interest (prevalent cases) and still living in Norway [16]. We calculated incidence rates per 1,000 individuals, overall and by age and sex.

### ADHD medication initiation in the year following diagnosis

We determined the proportion of individuals who initiated medication within 0–3 months (0–92 days) and 4–12 months (93–365 days) following their initial diagnosis, stratified by the year of diagnosis and age group. For these analyses we excluded individuals who had initiated medication before their diagnosis was registered or did not have an ADHD diagnosis recorded in NPR. Furthermore, we excluded those without a full year of follow-up, i.e. those diagnosed in 2024, and those who died or emigrated within the first year after diagnosis.

We described the proportion of newly diagnosed ADHD cases with medication initiation within 0–3 and 4–12 months of diagnosis, and characteristics of individuals by the timing of medication initiation.

We evaluated the influence of sociodemographic factors, on early initiation (0–3 months) and any initiation (0–12 months) compared with no initiation in those periods (later or never initiated) using logistic regression models. Independent variables in these models included age, sex, year of diagnosis, immigration status, birth order, child`s living situation, parental education, and region of birth (Table S1). Birth order was included as an indicator of family structure, a recognized sociodemographic determinant of ADHD diagnosis and treatment access in pediatric populations. Missing values for sociodemographic variables (< 5%) were imputed using multiple imputations by chained equations including all variables described in the study and 20 imputed datasets. Due to the very low proportion of missing data (< 5%), no sensitivity analysis without imputation was performed.

## Results

### Incidence of ADHD

**Fig. 2:**
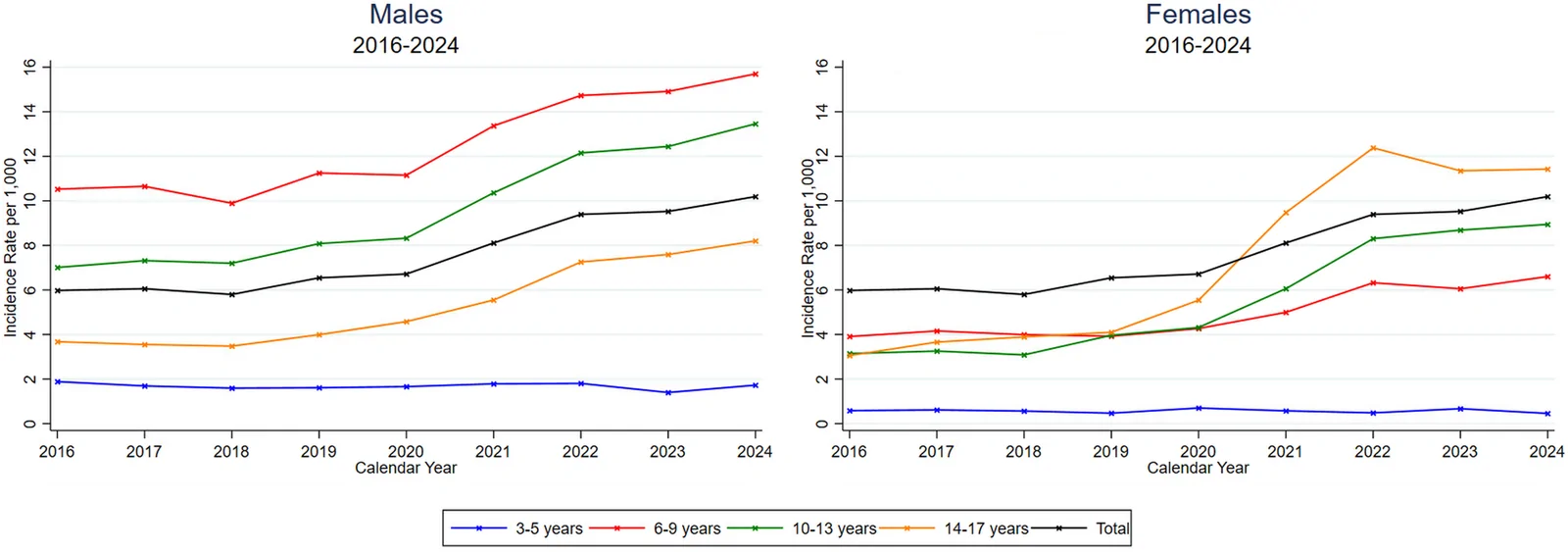
Time trends of incidence of ADHD diagnosis according to age and sex

Among 78,470 individuals with an ADHD diagnosis or dispensed ADHD medication at ages 3–17, we identified 52,149 individuals with incident ADHD from 2016 to 2024, with only 1547 (3%) identified by ADHD medication dispensing alone, and fewer than 4% were diagnosed before age 6 (Fig. 1). Overall, the incidence rate doubled from 4.4 to 9.0 per 1,000 individuals during the study period (Table S2). The increase was more pronounced and nearly quadrupled among 14-17-year-old females, while rates remained stable and low among 3-5-year-olds of both sexes (Fig. 2). The incidence rate was consistently higher in males below 14 years of age, but lower in 14-17-year-olds after 2016 (Table S2). In males, the highest incidence rates were consistently between ages 7 and 12 whereas these rates shifted to older ages in females during this period (Figure S1). Among females aged 14–17 years the incidence rate reached a peak of 12.4 per 1,000 in 2022 and subsequently remained at 11.4 per 1,000 in both 2023 and 2024.

### ADHD medication initiation

The overall proportion of patients initiating medication within one year after diagnosis increased from 72.8% in 2016 to 78.4% in 2023 (Table 1). Following a peak in 2021 (43.3%), the proportion of early initiation (0–3 months) declined, while the rate of delayed initiation (4–12 months) rose. By 2023, for the first time, more patients initiated medication in the delayed window compared to the early one (42.5% vs. 35.9%). Median days to medication initiation for incident ADHD patients ranged between 78 and 102 days from 2016 to 2023. The children aged 3–5 years consistently showed the lowest rates of early initiation. At the same time, delayed initiation has risen sharply in this group, among boys, from 24.9% in 2016 to 44.8% in 2023. By contrast, adolescents aged 14–17 years have the highest early initiation rates, approaching 50% for both sexes by 2021. Regarding sex, the differences between males and females within the same age groups are generally minor and inconsistent.

**Table 1:** Annual proportion of newly diagnosed patients initiating medication post diagnosis according to sex and age, *N* = 38,749

Year			2016	2017	2018	2019	2020	2021	2022	2023
Total newly diagnosed (N)			3,456	3,820	3,677	4,073	4,398	5,601	6,817	6,907
Median days to medication initiation			**93**	**78**	**87**	**81.5**	**85**	**81**	**89**	**102**
Proportion initiating within 0–3 months (%)			**35.9**	**40.3**	**36.9**	**40.1**	**41.3**	**43.3**	**40.2**	**35.9**
	**3–5 years**	Male	23.7	20.3	14.9	12.9	20.7	24.0	20.3	20.7
		Female	22.0	9.3	10.0	15.0	20.7	19.1	17.9	14.8
	**6–9 years**	Male	36.8	41.3	34.8	39.0	42.2	41.4	39.2	34.5
		Female	35.3	37.3	35.0	37.5	36.2	40.8	34.1	31.5
	**10–13 years**	Male	36.6	39.2	36.3	40.4	40.3	40.3	39.3	32.8
		Female	37.8	39.5	38.6	43.0	40.9	44.5	37.5	35.5
	**14–17 years**	Male	36.5	49.1	48.8	47.9	44.3	49.9	44.3	39.1
		Female	38.2	48.2	46.7	46.8	51.1	50.6	48.4	44.4
Proportion initiating within 4–12 months (%)			**36.9**	**31.2**	**33.2**	**33.3**	**35.5**	**35.9**	**38.3**	**42.5**
	**3–5 years**	Male	24.9	23.6	29.1	31.7	28.0	34.7	43.1	44.8
		Female	32.0	31.5	34.0	32.5	24.1	44.7	33.3	38.9
	**6–9 years**	Male	39.7	33.7	35.6	34.8	36.6	37.9	38.4	44.2
		Female	37.2	33.5	37.0	34.1	38.2	36.5	41.5	46.0
	**10–13 years**	Male	35.7	29.7	32.9	34.1	36.7	38.5	39.6	43.9
		Female	37.8	31.8	34.0	30.9	37.0	36.0	41.9	42.1
	**14–17 years**	Male	33.2	26.9	23.2	32.0	33.4	30.9	34.5	37.9
		Female	39.3	28.6	31.1	30.0	31.9	31.8	34.1	39.8
Proportion initiated within 0–12 months (%)			**72.8**	**71.5**	**70.1**	**73.3**	**76.8**	**79.1**	**78.5**	**78.4**

As shown in Table 2 the proportion of children with an immigrant background was lowest among early initiators (2.1%) and nearly twice as high in the no initiation group (3.9%). Similarly, children with at least one parent born in Africa or Asia were two to three times more common among non-initiators compared to the other groups. Parental education showed an inverse relationship with delayed initiation. Children initiating ADHD medication before a diagnosis was registered in NPR (suggesting possible initial diagnosis in private healthcare) had the highest proportion of mothers with higher education (46.8%), while the no initiation group had the highest proportion of mothers with low education (22.9%).

**Table 2:** Demographic characteristics by timing of ADHD medication initiation in newly diagnosed patients 2016–2023, *N* = 42,316

	Before diagnosis (*n* = 3,567)	0–3 months after (*n* = 15,226)	4–12 months after (*n* = 14,154)	No initiation within 12 months (*n* = 9,369)
Age; mean (SD)	11.1 (3.3)	11.0 (3.4)	10.5 (3.3)	10.2 (3.5)
Sex; n (%)				
Male	2,208 (61.9)	9,281 (61.0)	8,812 (62.3)	6,034 (64.4)
Female	1,359 (38.1)	5,945 (39.0)	5,342 (37.7)	3,335 (35.6)
Age category; n (%)				
3–5 years	75 (2.1)	297 (2.0)	510 (3.6)	766 (8.2)
6–9 years	1,261 (35.4)	5,827 (38.3)	5,858 (41.4)	3,709 (39.6)
10–13 years	1,210 (33.9)	4,785 (31.4)	4,661 (32.9)	2,993 (31.9)
14–17 years	1,021 (28.6)	4,317 (28.4)	3,125 (22.1)	1,901 (20.3)
Immigration status; n (%)				
Norwegian	3,479 (97.5)	14,901 (97.9)	13,819 (97.6)	9,003 (96.1)
Immigrants	88 (2.5)	325 (2.1)	335 (2.4)	366 (3.9)
Birth order; n (%)				
First	1,712 (48.0)	7,025 (46.1)	6,732 (47.6)	4,467 (47.7)
Second	1,234 (34.6)	5,350 (35.1)	4,902 (34.6)	3,154 (33.7)
Third	454 (12.7)	2,107 (13.8)	1,900 (13.4)	1,263 (13.5)
Fourth or higher	156 (4.4)	727 (4.8)	601 (4.2)	467 (5.0)
Missing	11 (0.3)	17 (0.1)	19 (0.1)	18 (0.2)
First medication; n (%)				
Methylphenidate	3,344 (93.7)	14,629 (96.1)	13,728 (97.0)	-
Atomoxetine	85 (2.4)	278 (1.8)	225 (1.5)	-
Lisdexamphetamine	131 (3.7)	301 (2.0)	182 (1.2)	-
Guanfacine	< 5	< 10	12 (0.1)	-
Amphetamine	< 5	< 5	< 5	-
Dexamphetamine	< 5	10 (0.1)	< 10	-
Child’s region of birth; n (%)				
Norway	3,382 (94.8)	14,546 (95.5)	13,532 (95.6)	8,803 (94.0)
Africa	20 (0.6)	57 (0.4)	60 (0.4)	58 (0.6)
Europe	94 (2.6)	328 (2.2)	331 (2.3)	281 (3.0)
Asia	42 (1.2)	177 (1.2)	130 (0.9)	151 (1.6)
America and Oceania	29 (0.8)	118 (0.8)	100 (0.7)	74 (0.8)
Missing	0 (0.0)	0 (0.0)	1 (0.0)	2 (0.0)
Child’s living situation; n (%)				
Two parents (married or cohabiting)	2,059 (57.7)	8,865 (58.2)	8,302 (58.7)	5,399 (57.6)
Others	1,334 (37.4)	5,784 (38.0)	5,364 (37.9)	3,670 (39.2)
Missing	174 (4.9)	577 (3.8)	488 (3.4)	300 (3.2)
Parental characteristics				
Mother’s education; n (%)				
≤ 10 years	705 (19.8)	3,128 (20.5)	2,949 (20.8)	2,149 (22.9)
11–13 years	1,152 (32.3)	5,121 (33.6)	4,801 (33.9)	3,011 (32.1)
≥ 14 years	1,670 (46.8)	6,833 (44.9)	6,262 (44.2)	4,059 (43.3)
Missing	40 (1.1)	144 (0.9)	142 (1.0)	150 (1.6)
Father’s education; n (%)				
≤ 10 years	868 (24.3)	3,670 (24.1)	3,407 (24.1)	2,230 (23.8)
11–13 years	1,532 (42.9)	7,003 (46.0)	6,668 (47.1)	4,126 (44.0)
≥ 14 years	1,011 (28.3)	4,016 (26.4)	3,575 (25.3)	2,506 (26.7)
Missing	156 (4.4)	537 (3.5)	504 (3.6)	507 (5.4)
Mother’s region of birth; n (%)				
Norway	3,104 (87.0)	13,476 (88.5)	12,490 (88.2)	7,552 (80.6)
Africa	57 (1.6)	176 (1.2)	206 (1.5)	306 (3.3)
Europe	234 (6.6)	871 (5.7)	847 (6.0)	805 (8.6)
Asia	103 (2.9)	451 (3.0)	394 (2.8)	486 (5.2)
America and Oceania	57 (1.6)	223 (1.5)	191 (1.3)	197 (2.1)
Missing	12 (0.3)	29 (0.2)	26 (0.2)	23 (0.2)
Father’s region of birth; n (%)				
Norway	3,007 (84.3)	13,080 (85.9)	12,188 (86.1)	7,342 (78.4)
Africa	74 (2.1)	202 (1.3)	233 (1.6)	321 (3.4)
Europe	223 (6.3)	906 (6.0)	887 (6.3)	783 (8.4)
Asia	92 (2.6)	407 (2.7)	303 (2.1)	422 (4.5)
America and Oceania	61 (1.7)	241 (1.6)	200 (1.4)	167 (1.8)
Missing	110 (3.1)	390 (2.6)	343 (2.4)	334 (3.6)

Young age at diagnosis (3–5 years) was associated with lower odds of early ADHD medication initiation, while older age at diagnosis (14–17 years) was associated with higher odds (Table 3). Immigrant children had significantly lower odds of early initiation compared to Norwegian-born children (univariate OR 0.7, 95% CI 0.6–0.8; multivariate OR 0.8, 95% CI 0.6–0.9). Children whose mother or father was born in Africa, Europe, or Asia showed lower odds of early initiation, particularly for parents born in Africa. The association with age at diagnosis showed the same pattern but weaker association for any initiation as compared to early initiation. For any initiation, immigrant children had lower odds of initiation in univariate but not multivariate analysis. Patterns for parental region of birth were similarly more pronounced in the univariable analysis, but odds of any medication initiation remained lower for children of parents born in Africa, Europe, and Asia. Lower paternal education was associated with higher odds of any initiation. In our cohort the results show that sex, birth order, and living situation do not play a significant role in medication initiation.

**Table 3:** Association between sociodemographic factors and initiation of ADHD medication in newly diagnosed ADHD patients, *N* = 38,749

	Early initiation (0–3 months)			Any initiation (0–12 months)	
	Univariable OR (95% CI)	Multivariable OR (95% CI)		Univariable OR (95% CI)	Multivariable OR (95% CI)
Sex					
Male	0.9 (0.9–0.9)		1.0 (0.9–1.5)	0.9 (0.8–0.9)	0.9 (0.9-1.0)
Female	Ref				
Year of diagnosis					
2016	Ref				
2017	1.2 (1.1–1.3)		1.2 (1.1–1.3)	0.9 (0.8-1.0)	0.9 (0.8-1.0)
2018	1.0 (0.9–1.2)		1.0 (0.9–1.1)	0.9 (0.8–0.9)	0.9 (0.8–0.9)
2019	1.2 (1.1–1.3)		1.2 (1.1–1.3)	1.0 (0.9–1.1)	1.0 (0.9–1.1)
2020	1.3 (1.2–1.4)		1.2 (1.1–1.3)	1.2 (1.1–1.3)	1.2 (1.1–1.4)
2021	1.4 (1.3–1.5)		1.3 (1.2–1.4)	1.4 (1.3–1.6)	1.4 (1.2–1.5)
2022	1.2 (1.1–1.3)		1.1 (1.0-1.2)	1.4 (1.2–1.5)	1.9 (1.2–1.9)
2023	1.0 (0.9–1.1)		0.9 (0.9-1.0)	1.4 (1.2–1.5)	1.3 (1.2–1.4)
Immigration status					
Native	Ref				
Immigrants	0.7 (0.6–0.8)		0.8 (0.6–0.9)	0.6 (0.5–0.6)	0.9 (0.8–1.2)
Age category					
3–5 years	0.4 (0.3–0.4)		0.4 (0.3–0.4)	0.3 (0.3–0.3)	0.4 (0.3–0.4)
6–9 years	Ref				
10–13 years	1.0 (0.9–1.1)		1.0 (0.9–1.1)	1.0 (0.9-1.0)	0.9 (0.9-1.0)
14–17 years	1.4 (1.3–1.5)		1.4 (1.3–1.6)	1.2 (1.2–1.3)	1.2 (1.1–1.3)
Birth order					
First	Ref				
Second	1.1 (1.0-1.1)		1.0 (0.9–1.1)	1.1 (1.0-1.1)	1.0 (0.9–1.1)
Third	1.1 (0.9–1.1)		1.0 (0.9–1.1)	1.0 (0.9–1.1)	1.0 (0.9–1.1)
Fourth or higher	1.1 (0.9–1.2)		1.1 (0.9–1.2)	0.9 (0.8-1.0)	0.9 (0.9–1.1)
Child’s region of birth					
Norway	Ref				
Africa	0.7 (0.5-1.0)		1.2 (0.8–1.7)	0.6 (0.5–0.9)	1.2 (0.8–1.8)
Europe	0.8 (0.7–0.9)		1.1 (0.8–1.3)	0.7 (0.6–0.8)	1.1 (0.9–1.4)
Asia	0.9 (0.8–1.2)		1.2 (0.9–1.5)	0.6 (0.5–0.8)	0.9 (0.8–1.3)
America and Oceania	1.0 (0.8–1.3)		1.1 (0.8–1.4)	0.9 (0.7–1.2)	1.2 (0.9–1.6)
Child’s living situation					
Two parents married or cohabiting	Ref				
Others	0.9 (0.9-1.0)		0.9 (0.9–0.9)	0.9 (0.9-1.0)	0.9 (0.9–0.9)
Mother’s region of birth					
Norway	Ref				
Africa	0.5 (0.4–0.6)		0.7 (0.5–0.9)	0.4 (0.3–0.4)	0.6 (0.4–0.7)
Europe	0.8 (0.7–0.9)		0.9 (0.7–0.9)	0.6 (0.6–0.7)	0.7 (0.6–0.8)
Asia	0.7 (0.7–0.9)		0.8 (0.6–0.9)	0.5 (0.5–0.6)	0.6 (0.5–0.7)
America and Oceania	0.8 (0.7-1.0)		0.9 (0.7-1.0)	0.6 (0.2–0.6)	0.6 (0.5–0.7)
Father’s region of birth					
Norway	Ref				
Africa	0.5 (0.5–0.6)		0.7 (0.5–0.9)	0.4 (0.3–0.5)	0.6 (0.4–0.7)
Europe	0.8 (0.7–0.9)		0.9 (0.7–0.9)	0.7 (0.6–0.7)	0.8 (0.7–0.8)
Asia	0.8 (0.7–0.9)		0.9 (0.8–1.1)	0.5 (0.4–0.6)	0.7 (0.6–0.8)
America and Oceania	0.9 (0.8–1.1)		0.9 (0.8–1.2)	0.7 (0.6–0.9)	0.8 (0.7–0.9)
Mother’s education					
≤ 10 years	0.9 (0.9–0.9)		1.0 (0.9-1.0)	0.9 (0.8–0.9)	0.9 (0.9-1.0)
11–13 years	0.9 (0.9-1.0)		1.0 (0.9-1.0)	1.0 (0.9–1.1)	1.0 (0.9–1.1)
≥ 14 years	Ref				
Father’s education					
≤ 10 years	0.9 (0.9-1.0)		1.1 (0.9–1.1)	1.0 (0.9–1.1)	1.2 (1.1–1.3)
11–13 years	0.9 (0.9-1.0)		1.0 (0.9–1.1)	1.1 (1.0-1.2)	1.1 (1.0-1.2)
≥ 14 years	Ref				

*CI* confidence interval, *OR* odds ratio, *Ref* reference group

## Discussion

This study aimed to describe trends in incident ADHD diagnoses among Norwegian children and adolescents (3–17 years) from 2016 to 2024 and to investigate medication initiation and its links to sociodemographic factors. The study shows that the overall incidence of diagnosis more than doubled from 4.4 to 9.0 per 1,000 in 3-17-year-olds during the study period. The most prominent change was a 3.7-fold increase in incidence among 14-17-years old females (from 3.1 to 11.4 per 1,000), which narrowed the sex gap in adolescents. The overall proportion of children initiating medication within one year of diagnosis rose only slightly from 73% to 78%, and this was driven by an increase in delayed initiation (4–12 months). Early initiation was strongly linked to age at diagnosis; adolescents (14–17 years) had high and increasing rates of early medication initiation. Furthermore, parental background was a key determinant along with father’s education, as children whose mothers were born in Africa or Asia, had significantly lower odds of medication initiation and if fathers had low education, then there were higher chances of medication initiation.

### Incidence rate

One explanation for the increase in diagnosis might be that the diagnostic criteria have been refined to better identify the condition in its various presentations. Although Norway’s official diagnostic system is ICD-10, the Norwegian national guidelines recommend using DSM-5 criteria when assessing ADHD [17]. The DSM-5 was released in 2013 and adopted for clinical use in Norway in 2021 [16, 17]. DSM-IV requirement that symptoms must be present before age seven was changed to age 12 in the DSM-5. For the first time, the DSM-5 included examples of how symptoms might appear in adolescents (e.g., “often has difficulty waiting his or her turn” could manifest as extreme impatience in traffic or in waiting in line) [18].

A narrowing of the sex gap in ADHD diagnoses has been observed among 14–17-year-olds, with a notable increase in diagnoses for females. This trend might be attributed to various factors, including the later recognition of the inattentive presentation of ADHD [19], which is more common in females. Increased mental health content on social media platforms might be linked to greater awareness [20], which may help some individuals better describe their symptoms to clinicians. Research shows that hormonal fluctuations actively exacerbate ADHD symptoms and mood disturbances. Furthermore, that individuals with ADHD are more vulnerable to comorbid conditions like PMDD and postpartum depression during these periods, highlighting a critical interaction between hormones and ADHD. A key finding is the significant gap in tailored treatments despite this known impact [21]. The rising diagnosis rates in 14–17-year-old females might be due to a catch-up effect, as individuals with less overt symptoms are being identified at later age, whereas males with hyperactive symptoms could have been diagnosed earlier in childhood.

### Medication initiation

The observed changes in ADHD medication initiation patterns from 2016 to 2023 signify a clinical shift in treatment. The overall increase in the proportion of patients initiating medication within 12 months of diagnosis (from 72.8% to 78.4%) reflects a slight increase in reliance on pharmacotherapy [22, 23]. It may also, however, reflect a lack of effective psychosocial interventions together with increased societal expectations, resulting in medication becoming the principal intervention available to improve functioning.

Our findings regarding the rise in ADHD incidence rate are coherent with previous studies from the Nordic region [4, 24, 25]. A Danish study from 2010 to 2020 revealed a U-shaped trend in the incidence of ADHD medication initiation, with prevalence steadily increasing in adolescents, with treatment initiation in 67% of children (age 6–12 years) and 53% of adolescents (age 13–17 years) post diagnosis in 2020 [25]. A Finnish study (2008–2019) reported a significant increase in new medication users, noting a strong male predominance though with a steep 7.6-fold rise in females and the highest incidence among 6–8-year-old males [24]. Comparing these trends, data from Norway (2016–2023) shows a similar overall increase in medication initiation but demonstrates a distinct pattern in sex disparity, where the difference between males and females has decreased over time, contrasting with the persistent male predominance highlighted in the Finnish study. Direct comparison with non-Nordic countries is methodologically constrained because few nations maintain comparable nationwide, longitudinal prescription registries with universal healthcare coverage, the Nordic countries function as a cohort for such research [14].

Our study shows a shift from early to delayed initiation of medication. This is strongly supported by guidelines [26, 27], which recommend a period of psychological education and behavioral intervention as a first-line treatment, before initiating medication. The influence of the child’s age is an important finding; 3–5 years olds are much less likely to be medicated because drugs are approved for treatment of ADHD for children aged six and above [27, 28]. Thus off-label medication used in the 3–5-year age group, which accounted for 14% of initiations, might be reserved for cases with severe ADHD. In accordance with treatment guidelines, methylphenidate was initiated in approximately 95% of cases [29]. Children aged 6–9 are less likely to initiate medication early and are the primary driver of the increase in delayed initiation. This pattern is in coherence with the Norwegian Health Directorate’s recommendations for nonpharmacological therapy before medication [27].

The observation that males often have slightly higher initiation rates at younger age is consistent with the treatment trends of ADHD [30]. However, the finding that adolescent females show initiation rates as high as or even exceeding their male counterparts is important. This likely reflects the delayed identification in females [31]. Their symptoms may be overlooked until the significant functional impairment is revealed. It is observed that adolescent females with ADHD are at high risk for internalizing disorders like anxiety and depression [32]. When these conditions are diagnosed, medication may be initiated, contributing to the elevated initiation rates in this group. This indicates that clinicians might be becoming more adept at recognizing and treating ADHD in females, for whom the presentation may have been previously missed [33].

The finding that adolescents (14–17 years) consistently show the highest rates of early medication initiation might be because of the unique challenges of this developmental stage. By adolescence, ADHD symptoms might have typically persisted for years, often leading to significant academic challenges, social rejection, and strained family relationships [34]. The initiation decision in adolescents can be complex, with high-stakes outcomes like driving safety, school completion, and college readiness on the line. Furthermore, literature indicates that untreated ADHD in adolescence is a significant risk factor for substance abuse, delinquency, and accidental injury [35, 36], justifying an urgent interventional approach.

The association between a later year of diagnosis (2020–2022) and higher odds of medication initiation (both early and any initiation) are likely to reflect evolving societal and systemic factors, increased public awareness and reduced stigma surrounding ADHD and mental health [33]. Changes in clinical guidelines from the Norwegian Directorate of Health which consistently endorse multimodal treatment, were likely implemented more consistently during these years [18]. The observed stabilization in 2023 could indicate a plateauing of the trend, potentially suggesting the clearance of a diagnostic backlog following updated diagnostic criteria (DSM-IV to DSM-5).

An important finding is the strong negative association between child’s immigrant status and region of birth virtually disappears after adjusting for parental region of birth and education. Immigrant families and those from minority backgrounds often face significant barriers to accessing care. These include language barriers, lack of familiarity with the host country’s healthcare system, and differing cultural conceptions of illness and treatment [37]. The univariable model captures this disadvantage. However, the multivariable model suggests that these barriers are not uniformly distributed but are concentrated among specific groups, particularly those with parents from non-Western European origins and potentially with low parental education. Once these factors are adjusted, the independent effect of being an immigrant child diminishes.

After accounting for parental education, children of parents born in Africa or Asia were less likely to begin medication. This is likely due to cultural factors, such as reluctance to seek help or concerns about psychiatric drugs [38]. Alternatively, there may be a genuine difference in how symptoms are presented or reported by parents from different cultural backgrounds, leading to diagnostic uncertainty and hesitancy to initiate medication [39]. In the multivariable logistic regression, the effect of low paternal education on medication initiation shows that children are more likely to initiate medication if their fathers have a lower level of education.

In our cohort sex, birth order, and living situation had only minor association to medication initiation. Specifically, the lack of a sex difference contradicts older studies where boys were more frequently treated [5], suggesting that in a Norwegian context, identification and treatment practices may have become more equitable. Whether a child is first-born or has older siblings has little effect on a family’s decision to initiate medication. Children in single-parent households were slightly less likely to initiate medication. This may be because single parents often face resource constraints, which are well-documented barriers to accessing and managing complex healthcare regimens [40].

A previous study reported an increase in the prevalence of ADHD medication use in Norway [4]. Among children aged 6 to 17 years, there was a two-fold increase in prevalence and incidence of use of ADHD medication in the period 2006–2022. While We found that the proportion of diagnosed children who started medication within 12 months increased slightly, while there was a large increase in new ADHD diagnoses. Thus, the principal driver of the observed rise in recorded ADHD medication use is likely increased diagnosis (possibly driven by changes in diagnostic practice, awareness, and guideline implementation), with increased medication initiation contributing to a lesser degree.

These findings have several implications for clinical practice. The increase in diagnoses among adolescent females suggests clinicians should maintain heightened awareness for the inattentive presentation in this group [19, 31, 33]. The shift toward delayed initiation in younger children supports continued emphasis on behavioral interventions before pharmacotherapy [26, 27]. The high rates of early initiation among adolescents may be justified given the functional impairments and risks of untreated ADHD in this developmental stage [34–36]. Finally, disparities by parental background indicate a need for culturally sensitive outreach to ensure equitable access to care [37, 38].

### Strengths and limitations

Our study has several important strengths. We have used data from Norwegian health registers for analysis, which cover the entire population and to which reporting is mandatory. We have used both diagnosis and medication data to identify the ADHD cases that make our analysis more complete than relying on diagnoses from public specialist healthcare alone. However, there are also some limitations to consider. We could not measure practical issues, like how easy it was to get a referral in different parts of the country. The Norwegian Patient Registry does not capture diagnoses from non-governmentally funded healthcare, or diagnoses outside Norway. However, our data suggests that few children are only diagnosed outside the public system. Registry data inherently lack the granularity to fully capture clinical complexity. Non-pharmacological treatments for ADHD (e.g., behavioral therapy, psychoeducation) are not available for analysis, limiting our ability to assess whether delayed medication initiation reflects active use of behavioral interventions.

## Conclusion

In conclusion, we observed an increase in incidence rate of ADHD diagnosis from 2016 to 2024, which coincided with updated diagnostic criteria and growing clinical and societal awareness. The proportion of newly diagnosed ADHD patients who initiated medication increased only slightly and shifted to > 3 months after initial diagnosis suggesting a changing clinical approach that appears more aligned with treatment guidelines. The findings also indicate that sociodemographic factors, such as age, immigration status, and paternal education level were associated with differences in treatment initiation, pointing to variations in healthcare access or perceptions. Overall, the rising incidence of ADHD medication use appears primarily related to increased diagnosis, with changes in medication practices representing a secondary, though notable, trend.

## Supplementary Information

Below is the link to the electronic supplementary material.

Supplementary Material 1

## Data Availability

The data used for this research cannot be shared by the authors. Others affiliated with a Norwegian research institution can apply to use similar linked data at helsedata.no, after obtaining ethical approval by a regional ethical committee, according to current legislation.
