# Supplementary material for: Incidence of ADHD diagnoses and medication initiation among children and adolescents in Norway from 2016-2024
Source: Eur Child Adolesc Psychiatry. 2026 Jun 29;35(11):3511–21. doi: 10.1007/s00787-026-03116-1 (PMC13710621; doi:10.1007/s00787-026-03116-1)
Supplement: Supplementary file 1 [file 787_2026_3116_MOESM1_ESM.docx]

**Incidence of ADHD diagnoses and medication initiation among children and adolescents in Norway from 2016-2024**

Saima Orangzeb,^1, 2,3^ Ingeborg Hartz,^1,2,4,^ Inger Johanne Bakken^1^, Ingvild Odsbu^1^, Kari Furu^1,5^, Svetlana Skurtveit ^1,3^, Per Normann Andersen^6^, Elisabeth Haug^2,7^, Jacqueline M. Cohen ^1,5^

Affiliations:

(1) Department of Chronic Diseases, Norwegian Institute of Public Health, Oslo, Norway

(2) Department of Research and Innovation, Innlandet Hospital Trust, Norway.

(3) Norwegian Centre for Addiction Research, University of Oslo

(4) Department of Health and Nursing Sciences, University of Inland Norway

(5) Centre for Fertility and Health, Norwegian Institute of Public Health, Oslo, Norway

(6) Department of Psychology, University of Inland Norway

(7) Department of Health Sciences, NTNU-Norwegian University of Science and Technology, Gjøvik

Corresponding author: Jacqueline M. Cohen

Email: Jacqueline.Cohen@fhi.no

Key words:

**Attention-Deficit/Hyperactivity Disorder, Incidence, Medication initiation, Pharmacotherapy, Norway, Children and adolescents, Registry-based study**

**Table S1. Variable list with source and description**

| **Variable** | **Categorization** | **Data Source** |
| --- | --- | --- |
| Birth year |  | NPR |
| Age at diagnosis | Calculated as first diagnosis date minus birth date continuous and categorized as  3-5 years old, 6-9 years old, 10-13 years old, and 14-17 years old. | NPR, SSB |
| Sex | Sex of the individual, categorized as male or female. | SSB |
| Immigration status | Immigration status of individuals as   - Immigrants - Native (born in Norway with two Norwegian born parents, born in Norway with immigrated parents, born abroad with one Norwegian born parent, born in Norway with one foreign parent, or born abroad with two Norwegian born parents) | SSB |
| Region of birth | - Norway - Europe - Asia - Americas + Oceania (North- and Central America, South America and Oceania) - Africa | SSB |
| Birth order | Birth order (First, second, third, fourth or higher) | SSB |
| Child`s living situation | - Two parents married or cohabiting - Others (only mother, mother + stepfather, only father, father + stepmother, and others) | SSB |
| **Parental characteristics** | | |
| Parental education (separately for mother and father) | - 10 years or less - 11-13 years - 14 years and above | SSB |
| Parental region of birth (separately for mother and father) | - Norway - Europe - Asia - America + Oceania (North- and Central America, South America and Oceania) - Africa | SSB |

NPR= Norwegian Patient Registry, SSB= Statistics Norway

**Table S2. Annual incidence of ADHD diagnoses**

| **Year** | **Population at Risk** | **Incident ADHD Cases** | **Incidence Rate per 1,000** | **Incidence Rate per 1,000** | | | | | | | |
| --- | --- | --- | --- | --- | --- | --- | --- | --- | --- | --- | --- |
|  |  |  |  | **3-5 years** | | **6-9 years** | | **10-13 years** | | **14-17 years** | |
|  |  |  |  | **Male** | **Female** | **Male** | **Female** | **Male** | **Female** | **Male** | **Female** |
| 2016 | 921,968 | 4,097 | 4.4 | 1.9 | 0.6 | 10.5 | 3.9 | 7.0 | 3.1 | 3.7 | 3.1 |
| 2017 | 925,582 | 4,278 | 4.6 | 1.7 | 0.6 | 10.7 | 4.2 | 7.3 | 3.3 | 3.6 | 3.7 |
| 2018 | 925,190 | 4,139 | 4.5 | 1.6 | 0.6 | 9.9 | 4.0 | 7.2 | 3.1 | 3.5 | 3.9 |
| 2019 | 922,098 | 4,595 | 5.0 | 1.6 | 0.5 | 11.3 | 3.9 | 8.1 | 4.0 | 4.0 | 4.1 |
| 2020 | 922,420 | 4,961 | 5.4 | 1.7 | 0.7 | 11.2 | 4.3 | 8.3 | 4.3 | 4.6 | 5.5 |
| 2021 | 918,611 | 6,380 | 6.9 | 1.8 | 0.6 | 13.4 | 5.0 | 10.4 | 6.1 | 5.5 | 9.5 |
| 2022 | 913,100 | 7,752 | 8.5 | 1.8 | 0.5 | 14.7 | 6.3 | 12.2 | 8.3 | 7.3 | 12.4 |
| 2023 | 915,845 | 7,768 | 8.5 | 1.4 | 0.7 | 14.9 | 6.1 | 12.4 | 8.7 | 7.6 | 11.4 |
| 2024 | 913,801 | 8,179 | 9.0 | 1.7 | 0.5 | 15.7 | 6.6 | 13.5 | 8.9 | 8.2 | 11.4 |

**Figure S1. Age and sex-specific ADHD incidence rates per 1,000 individuals ages 3-17**
